# Repetition enhancement and perceptual processing of visual word form

**DOI:** 10.3389/fnhum.2012.00206

**Published:** 2012-07-12

**Authors:** Karine Lebreton, Nicolas Villain, Gaël Chételat, Brigitte Landeau, Mohamed L. Seghier, François Lazeyras, Francis Eustache, Vicente Ibanez

**Affiliations:** ^1^INSERM, U1077Caen, France; ^2^Université de Caen Basse-Normandie, UMR-S1077Caen, France; ^3^Ecole Pratique des Hautes Etudes, UMR-S1077Caen, France; ^4^CHU de Caen, U1077Caen, France; ^5^Department of Radiology, University Hospitals of GenevaGeneva, Switzerland; ^6^Wellcome Trust Centre for Neuroimaging, Institute of Neurology, UCLLondon, UK; ^7^Psychiatric Neuroimaging Unit, Division of Neuropsychiatry, Department of Psychiatry, University Hospitals of GenevaGeneva, Switzerland

**Keywords:** priming, fMRI, repetition suppression, repetition enhancement, sharpening, visual processing

## Abstract

The current study investigated the cerebral basis of word perceptual repetition priming with fMRI during a letter detection task that manipulated the familiarity of perceptual word form and the number of repetitions. Some neuroimaging studies have reported increases, instead of decreases, in brain activations (called “repetition enhancement”) associated with repetition priming of unfamiliar stimuli which have been interpreted as the creation of new perceptual representations for unfamiliar items. According to this interpretation, several repetitions of unfamiliar items would then be necessary for the repetition priming to occur, a hypothesis not explicitly tested in prior studies. In the present study, using a letter detection task on briefly flashed words, we explored the effect of familiarity on brain response for word visual perceptual priming using both words with usual (i.e., familiar) and unusual (i.e., unfamiliar) font, presented up to four times for stimuli with unusual font. This allows potential changes in the brain responses for unfamiliar items to be assessed over several repetitions, i.e., repetition enhancement to suppression. Our results reveal significant increases of activity in the bilateral occipital areas related to repetition of words in both familiar and unfamiliar conditions. Our findings support the sharpening hypothesis, showing a lack of cerebral economy with repetition when the task requires the processing of all word features, whatever the familiarity of the material, and emphasize the influence of the nature of stimuli processing on its neuronal manifestation.

## Introduction

Priming is generally defined as an improvement or a change in the identification, production or classification of a stimulus as a result of a prior encounter with the same or a similar stimulus (Tulving and Schacter, 1990; Schacter and Buckner, 1998; Wiggs and Martin, 1998; Henson, 2003; Schacter et al., 2007). It represents an implicit form of memory which occurs even in the absence of conscious remembering and it may reflect a memory system distinct from explicit memory (Graf and Schacter, 1985; Gabrieli, 1998; Henson, 2003). From a multi-system memory point of view, two kinds of repetition priming have been distinguished, depending on two distinct memory systems: perceptual and conceptual (or semantic) priming. Perceptual priming (the focus of the present study) is based on the perceptual or physical representations of stimuli and is thought to depend on a perceptual representation system or perceptual memory, which stores and processes perceptual, but not semantic, features of items (Tulving and Schacter, 1990; Tulving, 1995; Eustache and Desgranges, 2008). Perceptual repetition priming is often assessed using identification tasks of degraded items or briefly flashed stimuli which are supposed to further solicit the perceptual memory (Brown et al., 1996; Berry et al., 1997; Lebreton et al., 2001; Gagnepain et al., 2008, 2011).

This repetition-based processing facilitation has been associated with reductions in neural activity when an experience is repeated. Several functional neuroimaging studies have consistently revealed a decrease in haemodynamic responses for repeated (primed) relative to unrepeated (unprimed) stimuli. This repetition-based neural change has been assigned numerous terms such as adaptation, repetition suppression or neural priming (for review see Grill-Spector et al., 2006), and has been observed within a set of specific cortical regions (Schacter et al., 1998, 2004, 2007; Schacter and Badgaiyan, 2001; Henson, 2003), depending on the type of stimulus (visual or auditory word, object, face, symbol…) and on the nature of representations across different stages of a processing stream (perceptual or conceptual). For example, repetition-related reductions associated with perceptual behavioral priming were reported in brain areas that are significantly active for novel items (or for the first presentation of item), and that are known to be involved in early perceptual processing. These reductions has been found in various visual areas (for a review, see Schacter et al., 2004) coding written words (e.g., Fiebach et al., 2005; Schott et al., 2006), faces (e.g., Eger et al., 2005), objects (e.g., Vuilleumier et al., 2002) or line drawings (e.g., Lebreton et al., 2001), and more recently in the auditory cortex during word listening paradigms (Gagnepain et al., 2008, 2011).

The precise neurophysiological mechanisms underlying reduced neural activity is unknown and the relationship between these neural and behavioral priming in human is still under debate (Schacter et al., 1998; Wiggs and Martin, 1998; Henson, 2003; Henson and Rugg, 2003; Maccotta and Buckner, 2004; Wig et al., 2005; Grill-Spector et al., 2006). Based on the neural phenomenon of “response suppression” which refers to decrease in the firing rate of neurons for repeated visual stimuli as demonstrated with single-cell recording in monkeys (Desimone, 1996), Wiggs and Martin (1998) postulated that “repetition suppression” reflects a “sharpening” or “tuning” mechanism related to behavioral priming. According to this model, during repetition, only neurons coding the discriminatory (or key) features of stimuli, relevant in distinguishing it from others stimuli, are reactivated, while neurons coding features unnecessary for processing that stimulus, i.e., unspecific features present in several stimuli, are not reactivated, resulting in the sharpening of this item's cortical representation. Thus, repetition suppression would reflect a partial reactivation of stimuli's features related to activity of fewer neurons and leads to decrease in the haemodynamic response. This more sparse or distinctive representation is thought to allow for more efficient stimulus processing and hence results in more accurate/faster behavioral responses.

On the other hand, some studies have also reported that repetition-related neural changes could depend on various factors such as the initial exposure duration (Zago et al., 2005; Voss and Gonsalves, 2010), the degree of overlap between the stimulus-specific processes engaged during the initial and subsequent presentations (Dobbins et al., 2004; Horner and Henson, 2008, 2011), the involvement of visual search (Kristjánsson et al., 2007; Kristjánsson and Campana, 2010), or the familiarity of the item. In particular, repetition increases rather than decreases of brain responses, called “repetition enhancement”, have been associated with repetition priming of unfamiliar stimuli such as novel 3D objects (Schacter et al., 1995), non-real objects (Soldan et al., 2008, 2010), unknown faces (Henson et al., 2000; Thiel et al., 2002), meaningless symbols (Henson et al., 2000) and pseudowords (Henson, 2001; Fiebach et al., 2005; Gagnepain et al., 2008, 2011). The component process model of priming proposed by Henson (2003) suggests that repetition enhancement for unfamiliar stimuli is a sign of the formation of new perceptual representations, an hypothesis that fits with the existence of a perceptual memory system that encodes, stores, and processes perceptual representations of stimuli. This hypothesis is mainly supported by neuroimaging studies that investigated the representation of meaningful vs. meaningless stimuli, while little is known about the repetition effect on familiar (or usual) compared to unfamiliar (or unusual) perceptual visual word form. According to this interpretation, repetition suppression would not play a role as long as stimuli do not have pre-existent perceptual representations in memory. However, this assumes that several repetitions of unfamiliar items are necessary for the occurrence of repetition suppression (Schacter et al., 1995). To date, this hypothesis has not been explicitly tested with unfamiliar stimuli. Taken together, the present fMRI study aimed at characterizing the neural changes associated with perceptual priming while manipulating (1) the familiarity of visual word form using usual and unusual font, and (2) the number of repetitions of words with unusual font.

## Materials and methods

### Participants

Twelve healthy right-handed subjects (7 females) aged from 20 to 30 years (mean: 26.1 ± 4.5 years) were recruited for the experiment. Participants were screened to exclude drug and/or alcohol abuse, neurological disorder and serious head injury, psychiatric illness, and contraindications to undergoing magnetic resonance imaging (MRI). Informed written consent was obtained from each volunteer prior to the scanning session. All procedures were approved by the local ethical committee and done in line with the declaration of Helsinki.

### Design and task

Before the main fMRI experiment, subjects undertook a behavioral testing session to determine the optimal tachistoscopic presentation time to be used in the main fMRI experiment. The presentation time allowing 80–90% of successful detection varied between 20 and 40 ms across subjects (29.5 ± 6.9 ms). This rapid subject-specific presentation time would facilitate behavioral priming (see “Introduction”).

**Figure 1:**
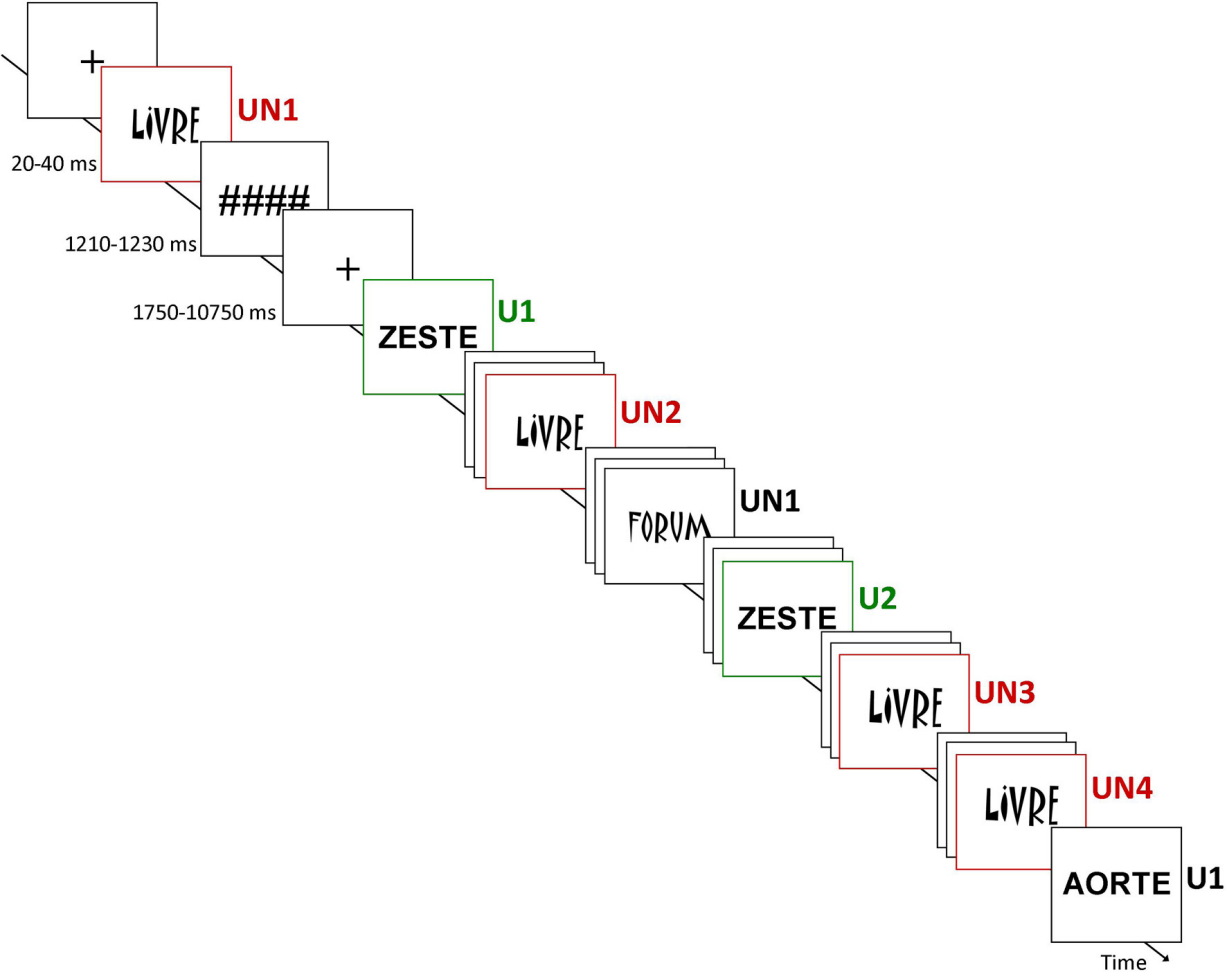
**Design of the study.** Subjects were instructed to detect whether the letter “A” was into the word. Unprimed items, corresponding to the 1st presentation of the word (U1 and UN1), and target items, corresponding to following presentation(s) of repeated words (U2, UN2, UN3, and UN4) were mixed together into each run. The familiarity of word perceptual form was manipulated using an usual (U) font (Abadi MT Condensed Light) and an unusual (UN) font (Matisse ITC). Presentations of words were preceded by a fixation cross (variable duration) and followed by a mask. The words in usual font were repeated twice (U1 and U2) while the words in unusual font were repeated four times (UN1, UN2, UN3, and UN4).

During the event-related fMRI experiment, the subjects had to detect the letter “A” into words by pressing a 2-button box (YES – NO) and to answer both quickly and accurately. Unprimed items, corresponding to words presented for the first time, and target items, corresponding to all subsequent presentations of the same words, were mixed together into each run. The words were selected from the Brulex French database (Content et al., 1990) and controlled for frequency (50–590 in 1,000,000) and length (5–7 letters). They were presented from 20 to 40 ms (see above), followed by a mask (#######), with a total time of presentation (word + mask) kept constant at 1250 ms, and a fixation cross varying from 1750 to 10250 ms (counterbalanced across conditions), independently of the fMRI volume acquisition (EPI MRI sequence TR). Two runs, of 7.4 min each, were performed. Into each run, words were presented in usual (U, Abadi MT Condensed Light) or unusual (UN, Matisse ITC) font (Figure 1). Note that the usual versus unusual nature of the font was decided a priori and did not correspond to the participant's judgment. One hundred and sixty words were presented once (U1, *n* = 4 × 20; UN1, *n* = 4 × 20); among them 80 were seen twice (U2, *n* = 4 × 10, inter-stimuli delay = 96.2 ± 20.3 s; UN2, *n* = 4 × 10, inter-stimuli delay = 94.7 ± 16.4 s) and 32 words with unusual font were seen four times (UN3, *n* = 4 × 8, inter-stimuli delay = 89.4 ± 20.1 s; UN4, *n* = 4 × 8, inter-stimuli delay = 93.7 ± 19.2 s). For each condition, half the words presented contained the letter “A” and the other half did not. Between each run, instructions were reminded to the subjects and feedbacks were recorded. After the scanning session, a debriefing was carried out individually to assess the cognitive strategy used by the subjects to perform the task, and notably whether they used conscious recollection of the repeated items.

### fMRI data acquisition

A 1.5 T Intera Philips MRI system at the Geneva University Hospitals (Geneva, Switzerland) was used to acquire both functional and anatomical images. EPI sequence was used to collect T2^*^-weighted images (TE = 40 ms, TR = 3000 ms, 30 axial slices, voxel size: 1.953 × 1.953 × 5 mm^3^). A structural T1-weighted scan was also obtained for each participant (TE = 5 ms, TR = 15 ms, flip angle = 30°, voxel size: 1.1 × 1.1 × 0.9 mm^3^).

Due to technical problems, entire dataset from one subject and one run from another were excluded. Therefore, subsequent analyses were conducted on 11 subjects (6 females; mean age: 25.8 ± 4.6 years).

### Data analysis

We analyzed behavioral priming effects in terms of both response time (RT) and percentage of correct responses (% CR) through repeated-measures ANOVA and paired t-tests. Incorrect responses were removed from RT data which were then trimmed to values within 2 standard deviations of the mean for each participant by deleting outlier values.

Regarding fMRI data: first, in order to check the lack of artifacts into the images, a variance volume was created for each subject's run and confirmed that most variability of the signal was restricted to the cortex. Moreover, we performed TSDiffana (http://imaging.mrc-cbu.cam.ac.uk/imaging/DataDiagnostics) on each functional raw volume acquired to ensure the lack of any isolated artefact into the images, leading to the exclusion of a single volume (among 6600). fMRI data processing and statistical analyzes were performed with the Statistical Parametric Mapping (SPM5) software package (Wellcome Trust Centre for Neuroimaging, London, UK) and the standard options set as default. For each subject, all functional volumes were first coregistered to the first one, and a mean functional image was obtained. Afterwards, the T1-weighted structural volume was coregistered to the mean functional image, and the coregistration accuracy was visually controlled for each subject individually. Between-slice timing differences induced by differences in acquisition order were then corrected. Normalization parameters, determined from the spatial normalization of the coregistered T1-weighted structural volume onto the standard T1 template from the Montreal Neurological Institute (MNI), were applied to each corresponding functional volume. The resultant normalized images were then smoothed with an isotropic 10 mm full width at half maximum gaussian kernel. Finally, the time series in each voxel were highpass-filtered to 1/128 Hz, scaled to a grand mean of 100, and averaged over all voxels and scans within a run.

fMRI data statistical analyses were conducted by using the general linear model on a voxelwise basis employing a random effects model implemented with a two level procedure. Only correct “A” detections were modeled as different experimental conditions (U1, U2, UN1, UN2, UN3, UN4), i.e., as δ functions at each stimulus onset. The ensuing haemodynamic response was modeled by convolving these δ functions with a canonical haemodynamic response function (HRF). Two fixed effect analyses on the main effect of the tasks (one for each type of material, i.e., words with usual and unusual fonts) were first performed in order to determine the brain regions involved in the task among our subjects. The resulting T-maps (*p* < 0.05 *Family Wise Error* corrected) were then used as inclusive explicit masks in all subsequent analyses, together with an anatomical mask including the bilateral occipital lobe (superior, middle and inferior occipital gyri, lingual gyri, cunei, calcarine sulci) and the fusiform gyri (from AAL labeling, Tzourio-Mazoyer et al., 2002). In other words, the intersection between the two anatomical and functional masks was used to constrain the analyses (9998 voxels = 79984 mm^3^). This is motivated by the fact that the BOLD visual perceptual priming is expected to involve visual brain regions (Henson, 2003; Schacter et al., 2007) and the repetition suppression phenomenon is, by definition, restricted to regions activated by the task (i.e., decrease in activations). Six simple contrasts were performed (i.e., U1 < U2, UN1 < UN2, UN1 < UN4, U1 > U2, UN1 > UN2, UN1 > UN4). In addition, in order to assess the effect of the four repetitions for words with unusual font, all the UN conditions were modeled as one single condition and the number of repetition was introduced as a parametric modulator (1, 2, 3, and 4 as parametric modulators). For each subject, the main effect of the parametric modulation was evaluated and then entered into a one-sample *t*-test for a second-level random effect analysis. Finally, we also evaluated the effect of behavioral facilitation by assessing the relationships between RT and brain activity. RTs were first transformed into *z*-scores (by subject, session, and type of answer) and then entered as a parametric modulator in the model.

Only clusters surviving a *p* < 0.005 uncorrected threshold as well as an extent threshold of 20 contiguous voxels were considered as significant in all analyses.

## Results

### Behavioral results

Data were reported in Table 1. The three 2 × 2 repeated-measures ANOVAs for the RT variable revealed neither any significant main effect of repetition [U1 vs. U2: *F*_(1, 10)_ = 1.16, *p* > 0.3; UN1 vs. UN2: *F*_(1, 10)_ = 0.19, *p* > 0.6; UN1 vs. UN4: *F*_(1, 10)_ = 0.19, *p* > 0.6] nor any significant interaction between repetition and type of answer [U1 vs. U2: *F*_(1, 10)_ = 0.23, *p* > 0.6; UN1 vs. UN2: *F*_(1, 10)_ = 0.96, *p* > 0.3; UN1 vs. UN4: *F*_(1, 10)_ = 2.30, *p* > 0.15]. However for each ANOVA, a significant main effect of type of answer [RTA < RTno-A: U1 vs. U2: *F*_(1, 10)_ = 6.04, *p* < 0.05; UN1 vs. UN2: *F*_(1, 10)_ = 13.04, *p* < 0.01; UN1 vs. UN4: *F*_(1, 10)_ = 7.96, *p* < 0.05] was found. The three paired *t*-test on the %CR did not reveal any significant effect of repetition [U1 vs. U2: *t* = 0.76, *p* > 0.4; UN1 vs. UN2: *t* = 1.19, *p* > 0.2; UN1 vs. UN4: *F*_(1, 10)_ = −1.53, *p* > 0.15].

**Table 1:** **Reaction times (ms) and % correct responses during the letter “A” detection task**.

	**Reaction time (ms)**		**%Correct response**
	**RT_A_**	**RT_no-A_**	
U1	794.7 ± 353.6	869.1 ± 306.9	88.2 ± 6.8
U2	783.6 ± 355.9	847.2 ± 298.9	86.8 ± 10.0
UN1	802.2 ± 347.2	909.3 ± 340.1	81.6 ± 10.7
UN2	803.7 ± 377.8	916.8 ± 325.8	77.4 ± 12.2
UN4	765.4 ± 372.3	895.0 ± 367.1	84.7 ± 11.4

U1 = 1st presentation of words with usual font; U2 = 2nd presentation of words with usual font; UN1 = 1st presentation of words with unusual font; UN4 = 4th presentation of words with unusual font.

The 4 × 2 repeated-measures ANOVA with RT variable for the four repetitions of words with unusual font revealed a significant main effect of type of answer [RT_A_ < RT_no−A_: *F*_(1, 10)_ = 13.48, *p* < 0.01] but the main effect of repetition [*F*_(3, 30)_ = 2.22, *p* > 0.1] and the interaction between the number of repetition and type of answer [*F*_(3, 30)_ = 1.92, *p* > 0.1] were not significant. The One-Way repeated-measures ANOVA (number of repetition) for the %CR did not reveal a significant effect of the number of repetition [*F*_(3, 30)_ = 1.68, *p* > 0.15].

The debriefing revealed that none of the subjects used encoding or retrieval memory processes to perform the letter detection task.

### fMRI data

**Figure 2:**
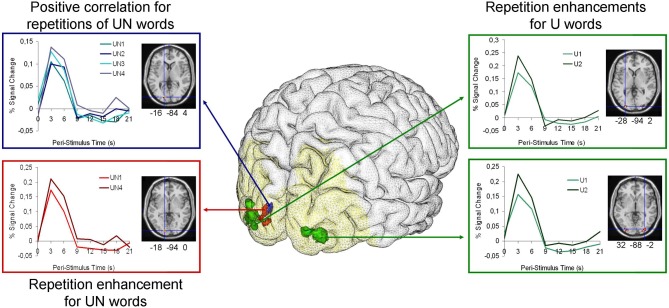
**Brain render with Anatomist (www.brainvisa.info) for each priming condition with the analysis mask (for words with usual font) in yellow and corresponding graphs of the haemodynamic time course data for each priming condition.** Green clusters represent the significant differences between the 2nd presentation of words in usual font (U2) and the 1st presentation of words in usual font (U1). The red cluster represents the significant differences between the 4th presentation of words in unusual font (UN4) and the 1st presentation of words in unusual font (UN1). The blue cluster shows the regions significantly correlated with the number of presentations of words in unusual font (UN). All clusters coordinates are given in the MNI space. Note that fMRI statistical analyses were performed on beta estimators of the BOLD response and not the peak of the hemodynamic response function shown here for the purpose of illustration.

The six simple contrasts revealed only BOLD responses increased for U2 vs. U1 condition in two clusters including the bilateral middle and inferior occipital gyri, the right calcarine sulcus and the left lingual gyrus (BA 18/19). No significant difference was found for the UN1 vs. UN2 contrasts (UN1 < UN2 or UN1 > UN2), but increases were also observed in a left cluster encompassing the superior and middle left occipital gyrus and left lingual gyrus (BA 17/18) for the UN4 vs. UN1 contrasts (UN4 < UN1 or UN4 > UN1) (Figure 2 and Table 2).

**Table 2:** **Brain areas involved in the different comparisons and correlations**.

**Region**	**Size (voxel/cm^3^)**	**MNI coordinates**			***Z*-Score**
		***x***	***y***	***z***	
**U2>U1**					
R Inferior Occipital Gyrus, BA 18/19	102/0.82	32	−88	−2	3.75
R Middle Occipital Gyrus, BA 18					
R Calcarine Sulcus, BA 18					
L Middle Occipital Gyrus, BA 18	122/0.98	−28	−94	2	3.60
L Inferior Occipital Gyrus, BA 18					
L Lingual Gyrus, BA 18					
**UN4>UN1**					
L Superior Occipital Gyrus, BA 17/18^*^	43/0.34	−18	−94	0	3.77
L Middle Occipital Gyrus, BA 18^*^					
L Lingual Gyrus, BA 18^*^					
**POSITIVE CORRELATION WITH UN REPETITIONS**					
L Superior Occipital Gyrus, BA 18^*^	24/0.19	−16	−84	4	3.47
L Calcarine Sulcus, BA 17^*^					

L = left; R = right; BA = Brodmann Area; U1 = 1st presentation of words with usual font; U2 = 2nd presentation of words with usual font; UN1 = 1st presentation of words with unusual font; UN4 = 4th presentation of words with unusual font;

* = at p < 0.01 uncorrected, the right counterparts were also involved.

In the parametric modulation analysis with the number of UN word repetitions, only positive correlations (i.e., increases in BOLD signal with repetition) were found in a left cluster that extended over the superior occipital gyrus and the calcarine sulcus (BA 17/18) (Figure 2 and Table 2).

Finally, the parametric modulation analysis with RT revealed only a negative correlation (i.e., increases in BOLD signal with decreases in RT) into several bilateral clusters including the bilateral calcarine sulci, the bilateral superior and inferior occipital gyri and the left middle occipital and lingual gyri (BA 17/18/19).

## Discussion

The present study highlights increases in brain activity associated with repetition for words written with both usual and unusual font. These increases mainly involved the bilateral BA18 areas, extending medially to the calcarine sulci and laterally to BA19. Moreover, despite a lack of significant behavioral facilitation, activity increases in these regions were positively related to the number of repetition and negatively to the RT for the word in unusual visual form (see Figure 2 and Table 2).

First, the anatomical sites of repetition-related brain activity increases in the present study are consistent with those of activity decreases reported in prior neuroimaging studies of word perceptual priming (Dehaene et al., 2001; Daselaar et al., 2005; Fiebach et al., 2005). Moreover, they encompass numerous regions involved in word processing, such as those belonging to the « left medial extrastriate cortex » cluster described in the metaanalysis of word processing studies by Jobard et al. (2003), but also their right counterparts (Hagoort et al., 1999; Fiebach et al., 2002). Thus, brain activity changes found in the present study occurred in brain regions dedicated to perceptual word processing. More precisely, the posterior localization of our results (BA 17/18/19) suggests the involvement of regions dedicated to low-level word processing (Jobard et al., 2003; Dehaene et al., 2005; Vinckier et al., 2007).

We found significant increases of activity in the occipital areas related to repetition of words in both familiar and unfamiliar conditions. Repetition enhancement for words with unusual font is consistent with some previous reports suggesting that this effect was likely a consequence of the unfamiliar nature of the material (Henson et al., 2000; Fiebach et al., 2005; Gagnepain et al., 2008, 2011). Nevertheless, it has also been observed here for words with familiar font suggesting the potential involvement of other factors.

Repetition enhancement for familiar material have previously been reported with particular conditions but these conditions did not fit with the present study: i.e., when items were not identified (James and Gauthier, 2006), when subjects did not pay attention to the items (Vuilleumier et al., 2005) or in a short-term priming experiment without mask between items (Schnyer et al., 2002). We argue that the repetition enhancement observed in the present study is related to the nature of the task processing. Indeed, processing isolated letters is not an usual step when words are treated as a whole, as suggested by the open bigrams model (Grainger and Van Heuven, 2003; Grainger and Whitney, 2004; Grainger et al., 2006). This model postulates that the detection of the relative position of two letters (bigram) into a word is the key step of word processing, and that bigrams are then combined, making the specificity of word. According to this model, the precise representation of an isolated letter into a word should not constitute a pre-existent perceptual representation. Therefore, in agreement with the component process model of priming proposed by Henson (2003 see “Introduction”), the repetition enhancement observed here might result from the creation of new perceptual representations of the letter “A” among words. The posterior location of this effect further reinforces this view. Indeed, the posterior regions showing repetition enhancement were assumed to be dedicated to low-level word-processing such as letter detection (e.g., Dehaene et al., 2005; Vinckier et al., 2007) and could therefore constitute the cerebral bases of the perceptual representation of a letter among words. In addition, this repetition enhancement was only observed in occipital regions after the fourth repetition for words in unusual font, while it was already observed after the second presentation for words in usual font. From a new perceptual representations formation point of view, it could be more difficult to create a new representation of the letter “A” among a word with unfamiliar font, due to the lack of prior visual perceptual representation of words in this unusual font. Consistent with this idea, our findings have also showed a significant negative correlation between these occipital activity increases and the quick and correct detection of the letter “A” into the words with the unusual font. Moreover, this relationship is unlikely to be related to specific, global, effects such as learning or attention change along runs as there was no significant effect of the position of items on RT. It is also unlikely due to the type of answer (RT_A_ < RT_no−A_) as this factor was taken into account when transforming RTs into *z*-scores.

The repetition enhancement associated with the repetition of words in familiar visual form here contrasts with previous studies that have reported significant reductions of cerebral activity in regions dedicated to word processing (Buckner et al., 1995; Schacter et al., 1996; Backman et al., 1997; Schacter et al., 1999; Buckner et al., 2000; Dehaene et al., 2001, 2004; Henson, 2001; Daselaar et al., 2005; Fiebach et al., 2005; Kouider et al., 2007; Ryan and Schnyer, 2007). According to the sharpening hypothesis (Wiggs and Martin, 1998), repetition suppression would reflect a reactivation of specific or discriminatory features of stimuli (see “Introduction”). This hypothesis also predicts that a perceptual task where subjects have to process all the stimuli's features (i.e., both specific and unspecific features) should induce a complete reactivation of stimuli's features during repetition and thus prevent repetition suppression. Several previous reports have indeed supported this prediction. More specifically, it is worth noting that in all priming studies where a significant repetition suppression was found, the task required a global processing of stimulus, in which only some features of items were essential to perform the task, while others could be ignored. For instance, in word-stem completion (Buckner et al., 1995, 2000; Schacter et al., 1996, 1999; Backman et al., 1997; Yasuno et al., 2000; Daselaar et al., 2005), lexical decision (Fiebach et al., 2005) and word reading tasks (Ryan and Schnyer, 2007) all word features (i.e., each letter) are not necessarily processed for the task to be completed. Similarly, tasks such as object recognition (Vuilleumier et al., 2005), item size judgments (Dobbins et al., 2004), object naming (Lebreton et al., 2001) and semantic decision tasks (Zago et al., 2005; Eddy et al., 2007) do not depend on the processing of each item feature. By contrast, the task used in the present study (i.e., letter detection) required an explicit processing of almost all the local word features. Thus, this task should have prevented any stimuli processing economy (sharpening), and therefore any repetition suppression, to occur. The present findings are thus consistent with the hypothesis of a lack of repetition suppression when the task does not allow for the economy of some feature processing. Besides, involvement of visual search processes could have limited the repetition suppression effect to occur. Indeed, our task (letter A detection into words) was effortful by inducing a new visual search for each stimulus display while Kristjánsson et al. (2007) have shown a repetition suppression effect when the visual search is eased (i.e., target position was repeated). Limited statistical power due to a small number of subjects is unlikely to account for the lack of repetition suppression since statistically significant increases were found in repetition-related occipital regions. Interestingly, the increase in brain activity was consistent across our different priming conditions (i.e., usual and unusual fonts) and statistical approaches (i.e., contrasts and correlations).

Altogether, our results are consistent with an increase of brain activity with repetition in absence of repetition suppression, whatever the familiarity of the material. These findings were interpreted according to the sharpening model of repetition priming—i.e., as a reflect of the reactivation of almost all features of stimuli, and the creation of new perceptual representations. We acknowledge below some other alternative interpretations.

First, the lack of behavioral effect could be the explanation for the lack of BOLD repetition suppression observed here. Although the relationship between behavioral priming and neural priming remains debated (e.g., Schacter et al., 1998; Wiggs and Martin, 1998; Henson, 2003; Henson and Rugg, 2003; Maccotta and Buckner, 2004; Wig et al., 2005; Grill-Spector et al., 2006), some authors assume that neural mechanisms are related to behavioral effects. In line with this assumption, our fMRI results could simply reflect the lack of behavioral priming. Thus, the unusual letter detection task involved in our experiment would have prevented any behavioral facilitation in time and/or accuracy, and therefore any BOLD repetition-suppression to occur. Nevertheless, as shown in James and Gauthier (2006), neural priming can be observed in the absence of behavioral priming. In addition, behavioral priming has already been observed parallel to fMRI repetition enhancement, e.g., the reduction of RT and BOLD repetition enhancement for repeated pseudowords (see Fiebach et al., 2005; Gagnepain et al., 2008, 2011). Finally, the magnitude of the behavioral repetition effect was low here, yielding to non-significant statistical differences when considering the small number of subjects that is typically used in neuroimaging studies, while BOLD repetition effects magnitude was higher, leading to significant repetition enhancement in the same group.

Besides, Turk-Browne et al. (2007) reported repetition enhancement related to stimuli with low visual quality. The tachistoscopic presentation of words followed by a mask used in the present study might correspond to such a situation as words were hard to identify (78–89% of correct “A” detection, depending on the font and the number of repetitions) and could thus be considered as perceptually degraded. However, this hypothesis cannot fully account for our findings since it does not explain the later repetition enhancement observed for words in unusual font compared to words with usual font (4th vs. 2nd presentation). To the contrary, in the light of this interpretation one would expect the reverse finding, i.e., higher repetition enhancement for more degraded, i.e., unusual, words (81% of correct “A” detection vs. 87% for usual words). Another potential interpretation arises from our current knowledge on brain visual processing, namely, cortical response to visual input is initially driven by coarse information and global aspects of the image. In fact, at ~250 ms from stimulus onset, the representation becomes fine-tuned and maximally stimulus-specific: each feature of object is represented optimally (Zago et al., 2005). Consequently, due to their limited presentation time (20–40 ms) in the present study, stimuli are unlikely to be fully fine-tuned at the end of their first presentation (see also Voss and Gonsalves, 2010). Furthermore, according to the latency or accumulation model of priming (Grill-Spector et al., 2006; James and Gauthier, 2006), the neural response to repeated items is earlier and faster than that of new items, and may thus be associated with more fine-tuned and more detailed cortical representations. Since more detailed cortical representation is associated with an increased occipital activity (e.g., Kouider et al., 2007), repetition enhancement in the present study could reflect enhanced fine-tuning due to faster cortical processing. Nonetheless, again, this interpretation fails to explain why the repetition enhancement observed here appears delayed for words with unusual font compared to words with usual font.

In summary, we highlighted here the important role of repetition enhancement in underlying repetition-related activation changes. We hypothesized that the increase in occipital activation might be the consequence of the nature of the task used in this study and especially the nature of word feature processing necessary to perform the task. Although this warrants further investigations, our findings have implication for understanding the neural basis of repetition effects and emphasize its diversity according to the way stimuli are processed.

### Conflict of interest statement

The authors declare that the research was conducted in the absence of any commercial or financial relationships that could be construed as a potential conflict on interest.
